# Assessing the associations between *Aedes* larval indices and dengue risk in Kalutara district, Sri Lanka: a hierarchical time series analysis from 2010 to 2019

**DOI:** 10.1186/s13071-022-05377-6

**Published:** 2022-08-03

**Authors:** Prasad Liyanage, Yesim Tozan, Hasitha Aravinda Tissera, Hans J. Overgaard, Joacim Rocklöv

**Affiliations:** 1 Department of Epidemiology and Global Health, Umeå University, Umeå, Sweden; 2 Ministry of Health, Colombo, Sri Lanka; 3 School of Global Public Health, New York University, New York, NY 10003 USA; 4 Faculty of Science and Technology, Norwegian University of Life Sciences, Ås, Norway; 5 Department of Microbiology, Faculty of Medicine, Khon Kaen University, Khon Kaen, Thailand; 6 Department of Public Health and Clinical Medicine, Section of Sustainable Health, Umeå University, SE-901 87 Umeå, Sweden; 7 Heidelberg Institute of Global Health & the Interdisciplinary Center for Scientific Computing, University of Heidelberg, Heidelberg, Germany

**Keywords:** Dengue risk, *Aedes* larval indices, Lags, Thresholds, Kalutara, Sri Lanka

## Abstract

**Background:**

Dengue is a major public health problem in Sri Lanka. *Aedes* vector surveillance and monitoring of larval indices are routine, long-established public health practices in the country. However, the association between *Aedes* larval indices and dengue incidence is poorly understood. It is crucial to evaluate lagged effects and threshold values of *Aedes* larval indices to set pragmatic targets for sustainable vector control interventions.

**Methods:**

Monthly *Aedes* larval indices and dengue cases in all 10 Medical Officer of Health (MOH) divisions in Kalutara district were obtained from 2010 to 2019. Using a novel statistical approach, a distributed lag non-linear model and a two-staged hierarchical meta-analysis, we estimated the overall non-linear and delayed effects of the Premise Index (PI), Breteau Index (BI) and Container Index (CI) on dengue incidence in Kalutara district. A set of MOH division-specific variables were evaluated within the same meta-analytical framework to determine their moderator effects on dengue risk. Using generalized additive models, we assessed the utility of *Aedes* larval indices in predicting dengue incidence.

**Results:**

We found that all three larval indices were associated with dengue risk at a lag of 1 to 2 months. The relationship between PI and dengue was homogeneous across MOH divisions, whereas that with BI and CI was heterogeneous. The threshold values of BI, PI and CI associated with dengue risk were 2, 15 and 45, respectively. All three indices showed a low to moderate accuracy in predicting dengue risk in Kalutara district.

**Conclusions:**

This study showed the potential of vector surveillance information in Kalutara district in developing a threshold-based, location-specific early warning system with a lead time of 2 months. The estimated thresholds are nonetheless time-bound and may not be universally applicable. Whenever longitudinal vector surveillance data areavailable, the methodological framework we propose here can be used to estimate location-specific *Aedes* larval index thresholds in any other dengue-endemic setting.

**Graphical Abstract:**

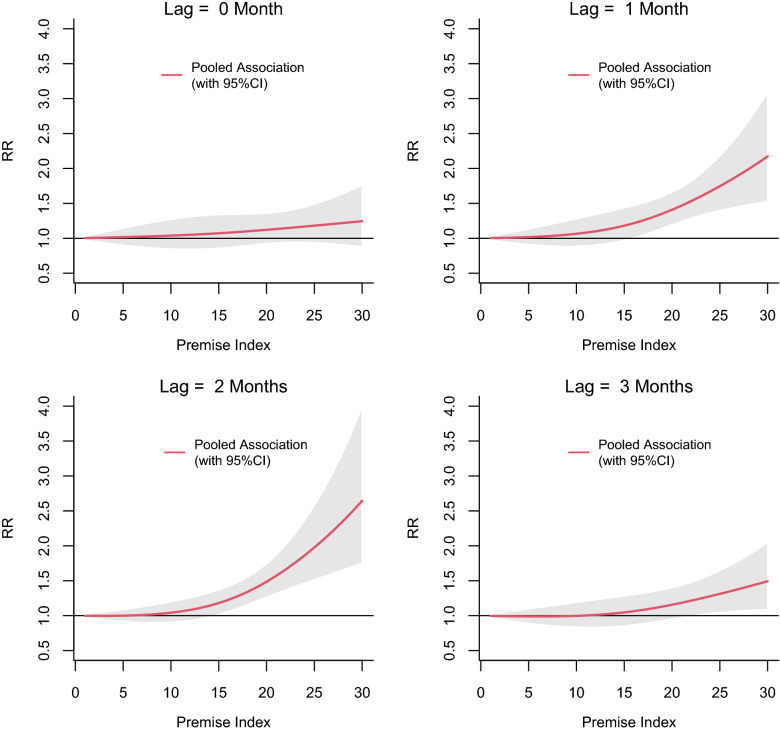

**Supplementary Information:**

The online version contains supplementary material available at 10.1186/s13071-022-05377-6.

## Background

Dengue is a rapidly spreading mosquito-borne viral disease with a substantial public health burden [1]. An estimated 390 million dengue infections occur annually, with 96 million people developing clinical manifestations [2]. Dengue virus (DENV) is transmitted by female *Aedes* mosquitoes, primarily by *Aedes aegypti* and secondarily by *Aedes albopictus* [3]. Dengue is endemic in Sri Lanka and was first serologically confirmed in 1962. The first island-wide outbreak occurred in 1965 [4]. Progressively larger inter-annual epidemics have been reported since the early 2000s. Today, dengue is the most significant public health problem among infectious diseases in Sri Lanka. Nearly 40% of the annual dengue cases are reported from Colombo, Gampaha and Kalutara districts in Western province [5]. The peak transmission period usually starts in June with the southwest Monsoon (SWM) from May to September, followed by a less severe peak in October–December with the second inter-monsoon (SIM). The social and environmental determinants of dengue transmission appear to vary across the country. Weather events, unplanned urbanization and inadequate garbage disposal and recycling services give rise to breeding sites and the proliferation of dengue vectors [6]. Even if an effective vaccine is available in the future, sustained vector control interventions will be needed to curtail the continuous spread of dengue due to increasing urbanization and climate change impacts [7].

The Stegomyia indices in use today were developed about 100 years ago. These indices are based on the degree of infestation by immature mosquitoes and serve as a proxy measure of adult vector densities [8]. Vector surveillance is an essential tool for assessing the spatial and temporal distribution of dengue vectors, predicting outbreaks in advance and assessing vector control interventions [9]. The earliest indices were the House Index (HI; in this article, the HI is called the Premise Index [PI]) and the Container Index (CI), defined as the percentage of houses infested with larvae or pupae and the percentage of water-holding containers infested with larvae or pupae, respectively [8]. The Breteau Index (BI), which was developed later, is defined as the number of positive containers per 100 premises [10, 11], and it considered a more informative vector index because it describes the number of positive containers in relation to the number of houses [10, 12, 13]. Indices that directly describe pupal and adult vector densities appear to be superior to larval indices in predicting dengue transmission [13–16]. An even better index of dengue risk is the presence of DENV-infected *Aedes* mosquitoes [17]. However, pupal and adult mosquito collections are both time-consuming and labor-intensive activities.

The lagged relationships between weather conditions and dengue outbreaks and incidence are well documented [18–20] and hold promise for the development of early warning and response systems. Preventive actions that target vectors are in the causal pathway, from weather conditions to dengue transmission. Further, vector surveillance information indicates the type and distribution of vector breeding places and guides prioritization and targeting of vector control interventions. It is, therefore, important to understand the delays from vector development to dengue transmission and the threshold values for vector indices to make evidence-based decisions for effective vector management [16]. In this context, we have previously reported the lagged associations between weather variables and dengue incidence on one hand [15], and weather variables and vector indices on the other hand [21] in Kalutara district in Western province of Sri Lanka. As shown by a systematic review conducted by Bowman et al., there is a knowledge gap in understanding the associations between *Aedes* larval indices and dengue transmission risk [16]. Several attempts have been made to address this research question, and there is an increasing body of evidence in support of the existence of such associations in dengue endemic settings with varying results [22–27]. However, the associations between vector indices and dengue incidence need to be further clarified as they might be unique to specific locations due to spatiotemporal variations and the already available evidence depends on data and methods used. The objective of the present study was threefold. Using a novel two-stage hierarchical approach, we first investigated the non-linear and delayed associations between *Aedes* vector indices and dengue incidence using PI, CI and BI across 10 sub-district units (Medical Officer of Health [MOH] divisions) in Kalutara district. We next assessed the influence of MOH division-specific factors on the relationship between vector indices and dengue incidence. Last, we compared the utility of each vector index in predicting dengue outbreaks.

## Methods

### Study setting

Kalutara district is situated adjacent to the southern border of Colombo, the central metropolitan area in Sri Lanka. The district's geographical boundaries fall within the latitudes of 6°47′ N and 6°91′ N and the longitudes of 79°570′ E and 80°18′ E. It spreads from the coastal area in the west to the edge of the mountain ranges and rainforests in the central part of the island. The altitude is < 150 m a.s.l. in most parts of the district. The district is divided into 10 MOH divisions, with a wide diversity in geographical characteristics, climate and population densities among these MOH divisions. Kalutara has a population of around 1 million over a land area of 1501 km^2^ [28]. The average population density is 662 (range 208–3352) persons/km^2^ across MOH divisions.

### Disease surveillance

Dengue was declared a notifiable disease under the national integrated surveillance system for communicable diseases in 1996. The system has island-wide coverage through trained clinical and public health staff [29]. The integrated surveillance system combines passive and enhanced sentinel surveillance methods and relies mainly on clinical diagnosis of dengue cases. According to a standard case definition for dengue, symptomatic patients are captured based on the 1997/2011 WHO classification [30–32]. In addition, a newly established online sentinel hospital-reporting system provides early warning for timely detection and mitigation of dengue outbreaks. Cases are notified to the MOH area where patients reside, triggering household-level control measures. For the present study, we extracted weekly dengue case count data for Kalutara district from the national integrated surveillance system.

### Vector surveillance

**Fig. 1:**
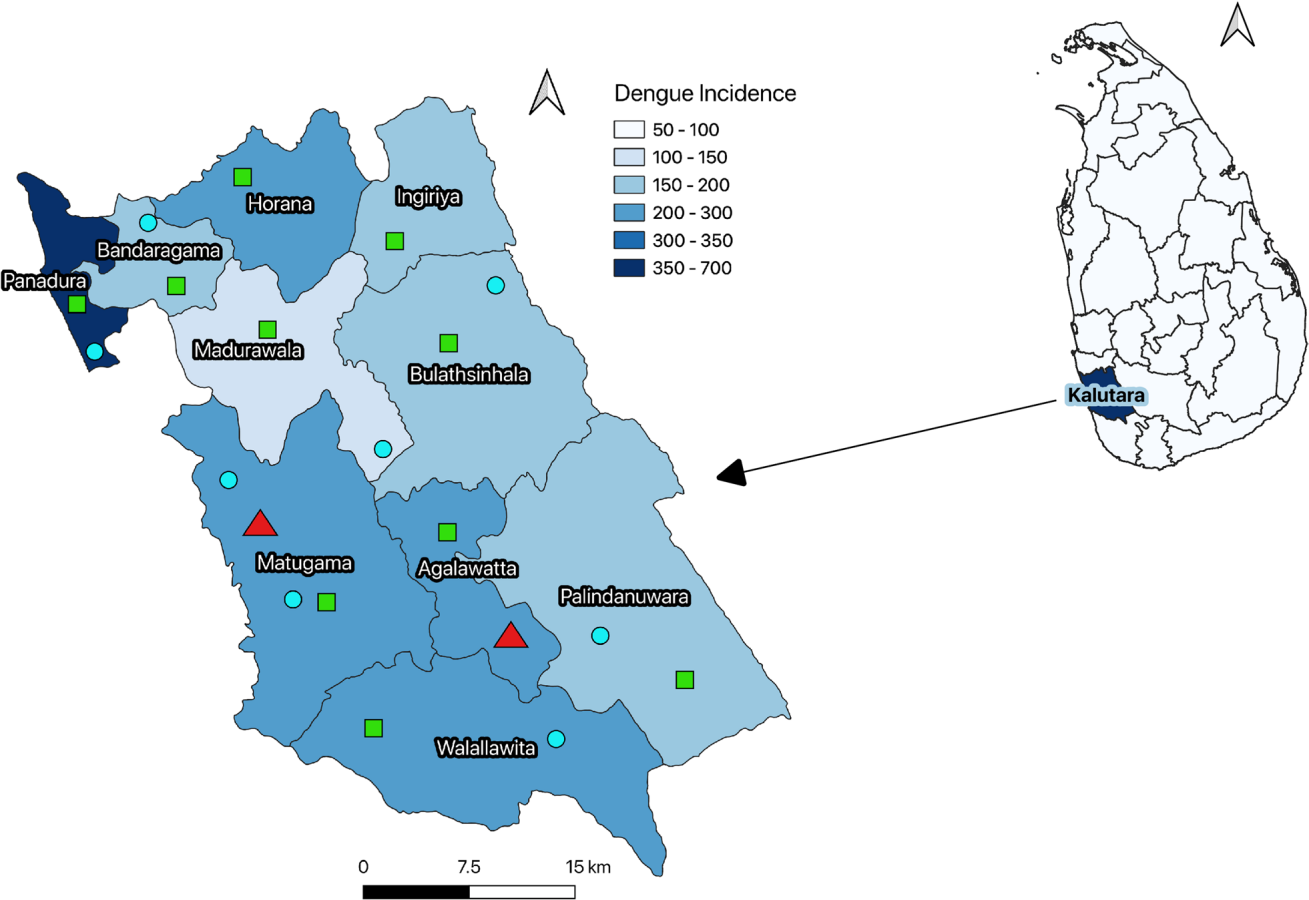
Dengue incidence and location of meteorological monitoring stations and entomological sentinel sites in Kalutara district, Sri Lanka. Black solid lines in the map represent the boundaries of Medical Officer of Health (MOH) divisions. Blue circles and red triangles show the location of rainfall and temperature monitoring stations, respectively. Green-shaded boxes indicate the location of long-term entomological surveillance sites. Annual dengue incidence was calculated per 100,000 population from 2010 to 2019. Source of the base file: https://data.humdata.org/dataset/sri-lanka-administrative-levels-0-4-boundaries

Dengue vector surveillance in Kalutara district has three components: (i) long-term sentinel site surveillance; (2) routine site surveillance; and (iii) sporadic vector surveillance in identified outbreak areas. In sentinel and routine site surveillance, entomological surveys are carried out systematically in pre-determined designated areas in the district. The “Grama Niladhari” (GN) divisions (smaller administrative units within each MOH division), where the most significant seasonal dengue outbreaks historically occurred, were selected as long-term sentinel or routine surveillance sites. Altogether, 10 such long-term surveillance sites were available in Kalutara district, distributed as one site per each MOH division (Fig. 1). Dengue vector surveillance is carried out by a team of Health Entomology Officers (HEOs) who are appointed to each district, with each team headed by a trained district entomologist. Entomological surveys are routinely conducted according to Sri Lanka’s national guidelines on *Aedes* vector surveillance and control under the technical supervision of the National Dengue Control Unit [33]. Ground-level and above-ground areas both indoors and outdoors are examined thoroughly to identify vector breeding sites. Teams use standard dipping, siphoning and pipetting methods to collect larvae [34]. A minimum of 100 randomly selected houses or premises in a sentinel and a routine site are surveyed at least once a month to observe trends in vector density. At the end of each survey, all mosquito species are identified, and the larval indices and the distribution and types of breeding sites for *Ae. albopictus* and *Ae. aegypti* are reported. In the present study in Kalutara district, *Ae. albopictus* was the most prevalent species, having a period prevalence of 97% of all positive containers over the study period; in contrast, the period prevalence of *Ae. aegypti* was only 3%. Due to the ubiquitous nature of the spatial distribution of *Ae. albopictus*, in our study, we used combined vector indices (presence of either *Ae. albopictus* or *Ae. aegypti* alone or together) to quantify the spatial risk of dengue. We used monthly values for the combined PI, BI, and CI from long-term sentinel and routine sites in MOH divisions from 2010 to 2019 collected and compiled at the office of the Regional Director of Health Service Kalutara.

### MOH division-specific characteristics

A set of 13 MOH division-specific characteristics that could affect vector density and dengue transmission were included in the analysis. These characteristics include land area, human population density, per-capita land use, household land use, number and type of houses (luxury houses, slums or huts), land area of rubber cultivation, number of manufacturing industries, number of schools, size of school-going population at each MOH division and related data. All data were extracted from the District Statistics Book published by the Department of Census and Statistics [35]. Rainfall and temperature data were obtained from eight rainfall and two temperature monitoring stations run by the Department of Meteorology. The geographic locations of weather monitoring stations and vector surveillance sites are shown in Fig. 1.

### Statistical analysis

A two-stage hierarchical procedure was used to examine the association between *Aedes* larval indices (i.e. PI, BI and CI) and dengue incidence in Kalutara district. In the first stage, non-linear lagged vector-to-dengue associations were derived for each MOH division using a quasi-Poisson time series regression model [36]. We used a distributed lag non-linear model (DLNM) and implemented it in the R package *dlnm* for this estimation [37]. The DLNM method employs the concept of cross-basis function to describe the delayed relationship between vector indices and dengue incidence flexibly. In the second stage, we combined the lagged vector-to-dengue associations for each MOH division in a hierarchical meta-analysis model to derive a pooled association. We used R package *mvmeta* for the meta-analysis [38]. Further, we examined the contribution of the MOH division-specific characteristics (Table 1) to the heterogeneity in the vector-dengue relationship in Kalutara district. All statistical analyses were done in the R statistical environment using R software version 4.1.0 [39].

**Table 1:** Cumulative dengue cases, annual average dengue incidence, and Medical Officer of Health division-specific variables averaged across all years in the study period in Kalutara District, Sri Lanka, 2010–2019

MOH division-specific characteristics	Long-term surveillance sites/MOH Division sites									
	Agalawatta	Bandaragama	Bulathsinhala	Horana	Ingiriya	Madurawala	Mathugama	Palindanuwara	Walallavita	Panadura
Cumulative dengue cases,* n*	1017	2549	1341	2822	1179	1079	3508	1173	1430	11,876
Annual average dengue incidence, * n* per 100,000	248	183	189	239	198	116	262	198	228	485
Premise Index, mean (range)	11.0 (0.9–24.6)	11.0 (1.6–63.3)	10.8 (1.9–24.0)	9.6(1.5–23.0)	10.8 (0.0–25.0)	10.9 (0.0–24.0)	10.4 (1.0 -29.0)	10.1 (0.0 -25.0)	10.6 (0.0 23.0)	6.6 (0.0- 24.0)
Breteau Index, mean (range)	13.4 (0.9–43.8)	12.4 (2.0–31.0)	14.7 (1.9 -64.0)	12.9 (1.9–38.0)	15.8 (0.0–54.0)	14.3 (0.0–40.3)	13.8 (1.0–41.6)	13.7 (0.0–36.0)	13.7 (0.0–36.0)	8.2 (0.0–26.8)
Container Index, mean (range)	26.5 (5.4–54.0)	26.4 (9.4–48.9)	22.5 (3.9–48.7)	20 (6.4–50.0)	19.6 (0.0–50.0	27.7 (0.0–60.5)	21.1 (3.8–50.0)	28.8 (0.0–57.6)	29.4 (0.0–69.5)	21.3 (0.0–66.6)
Population density (persons/km^2^)	415	2091	334	1078	640	665	507	208	279	3352
Land area (ha)	8800	5600	20,600	10,900	9000	14,000	26,400	27,600	20,900	7200
Per-capita land use (ha)	0.2	0.1	0.3	0.1	0.2	0.2	0.2	0.5	0.4	0.1
Household land use (ha)	0.9	0.2	1.2	0.4	0.6	0.7	0.6	2	1.4	0.1
Number of houses	9649	27,263	17,261	29,431	13,921	13,644	20,709	13,675	14,659	44,931
Number of luxury houses	0	10	1	14	0	17	11	4	0	303
Number of huts and shanties	6	29	31	84	77	80	102	53	12	144
Land area of rubber cultivation (ha)	370	654	14,614	5128	5128	6095	18,300	10,122	6172	145
Manufacturing industry,* n*	11	177	302	118	288	343	441	66	11	74
Number of schools	21	21	32	31	22	31	67	38	30	42
School-going population,* n*	5495	14,637	9269	30,289	7472	9329	32,355	8782	8310	39,417
Mean cumulative rainfall (mm/month)	337.1	241.2	367.8	241.2	348.1	357.5	259.8	273.6	352.8	215.6
Mean monthly temperature (°C)	31.8	30	31.8	31.8	31.8	30	30	31.8	31.8	30

*MOH* Medical Officer of Health

#### First-stage division-specific model

$$D_{i} \sim \, quasiPoisson\left( {\mu_{ti} } \right)$$

$$E\left( {D_{(ti)} } \right) \, = \, \beta_{i} + \, f\left( {LI_{ti} , \, vardf, \, lagdf} \right) \, + \, s\left( {T_{t} , \, timedf} \right) + log\left( {Population_{ti} } \right)$$

where *E(D*_*(ti)*_*)* is the expected number of dengue cases in month *t* in a MOH division denoted by *i*; *β* is the intercept in the MOH division *i*; *f(LI*_*ti*_*, vardf, lagdf)* is the cross-basis function for larval index in each MOH division *i* with corresponding degree of freedom (*vardf*) and its lagged association (*lagdf*); *s(T*_*t*_*,timedf)* is the smooth function of time with corresponding degree of freedom *timedf*. Monthly dengue cases were assumed to follow a quasi-Poisson distribution, which allows overdispersion [36]. Mid-year population in each MOH division was included in the model to adjust for changes in population growth and disparities in different sub-divisions over the decade. A detailed description of the definition of the cross-basis functions, adjustments for seasonality and trend, sensitivity analysis and model selection procedure are given in Additional file 1: Text S1; Table S1. Model diagnostic plots are given in Additional file 1: Figures. S1–S3.

#### Second-stage meta-analysis

The estimated associations at the MOH division level were pooled with a multivariate meta-analysis using the maximum likelihood approach [40]. We plotted the pooled lag-specific associations for Kalutara district at time lags of 0–3 months for each vector index. The heterogeneity in the associations across the MOH divisions was assessed using the Cochran Q-test of residual heterogeneity [41]. The proportion of total variation between divisions attributable to heterogeneity was further quantified by the related *I*^2^ index [42]. To examine if the heterogeneity observed could be explained in part by MOH division-specific characteristics (as given in Table 1) and to determine the influence of these characteristics on dengue risk, i.e. the moderating effects, we extended the second-stage analysis by regressing these variables in a univariable multivariate meta-regression framework provided in the *mvmeta* package [38]. The Akaike information criterion (AIC) was used to compare the fit of the models with each division-specific variable to its base model with no such variables introduced.

The statistical significance of the moderating effects of the division-specific variables was tested using the multivariate Wald test [38, 43]. All significance tests were done using an α value of 0.05 and 95% confidence limits for all variables. The moderating effects on the pooled vector-dengue relationship were predicted at values representing the 25th and 75th percentiles of the range of each division-specific variable. We plotted the sum of the pooled lag-specific associations for each division-specific variable analyzed at their 25th and 75th percentile values along with the overall cumulative associations (district average) without such moderating effects. We further assessed the direction of their moderating effects at the high end, i.e. at the 75th percentile. A detailed description is available in the Additional file 1: Text S2.

#### Evaluating the capacity of vector indices in predicting dengue outbreaks in Kalutara district

We evaluated the utility of each of the three larval indices in predicting dengue outbreaks using the generalized additive modeling framework. We converted the annual outbreak threshold of 100 cases per 100,000 population, which was operationally defined by the National Dengue Control Unit (NDCU), into monthly outbreak thresholds for each MOH division as a cutoff value for outbreak prediction. We used a two-staged hierarchical approach to evaluate the predictive performance of each vector index. The predictive abilities of each vector index were quantified for each MOH division for all outbreak years at the first stage. These MOH division-specific estimates were subsequently subjected to meta-analysis at the second stage to obtain the overall performance of each index. A detailed description of the model, the selection of the best performing lag combination and how the predictive performance of the models with the selected lag combination was evaluated using receiver operating characteristic curve (ROC) analysis are given in the Additional file 1: Text S3.

## Results

**Fig. 2:**
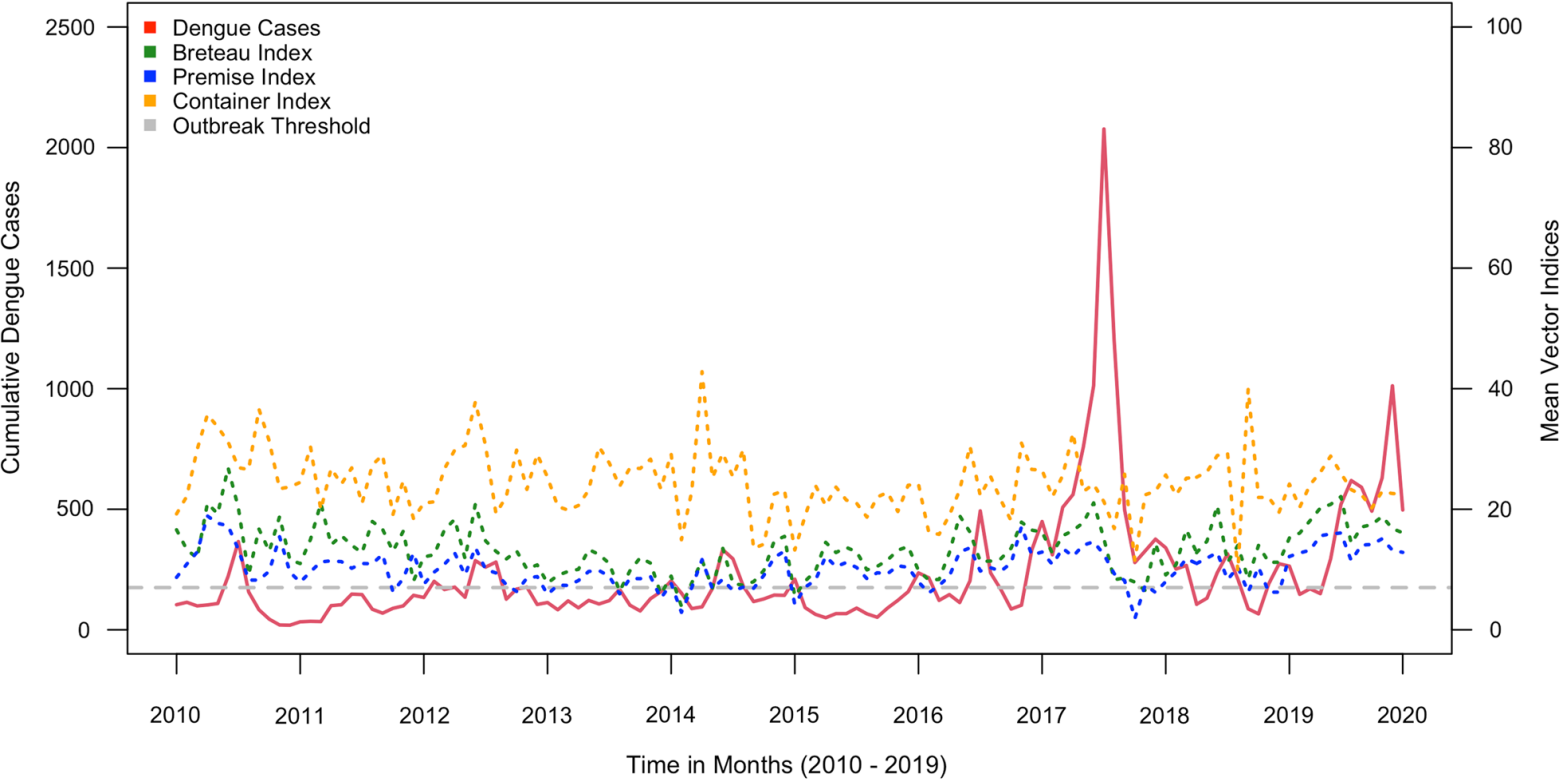
Time series plot shows the monthly aggregated dengue cases and average Premise Index (PI), Breteau Index (BI) and Container Index (CI) from 2010 to 2019 in all MOH areas in Kalutara district, Sri Lanka. The red line indicates the number of dengue cases; the green, blue and orange dotted lines indicate BI, PI, and CI; the horizontal dashed line indicates the outbreak threshold of 175 cases per month

Monthly cumulative dengue cases from 2010 to 2019 and the mean PI, BI and CI observed in corresponding months in all MOH divisions in Kalutara district are shown in Fig. 2. The total number of reported dengue cases during the study period was 27,974, and the outbreak threshold was 175 cases per 100,000 population per month. All three larval indices appeared to fluctuate constantly, giving rise to bi-annual peaks that coincided with monsoonal periods (SWM and SIM). The outbreak years were identified as 2010, 2012, 2014, 2016, 2017 and 2019. It was observed that, in all outbreak years, the seasonal surge in dengue cases in May and June was preceded by an increase in vector indices. The lowest dip in PI was observed in the latter part of 2017, and this reflected the impact of extensive vector control interventions implemented in response to the most significant dengue epidemic experienced in the country.

The annual dengue incidence and the MOH division-specific variables for Kalutara district were averaged over the study period (2010—2019) and are presented in Table 1.

**Fig. 3:**
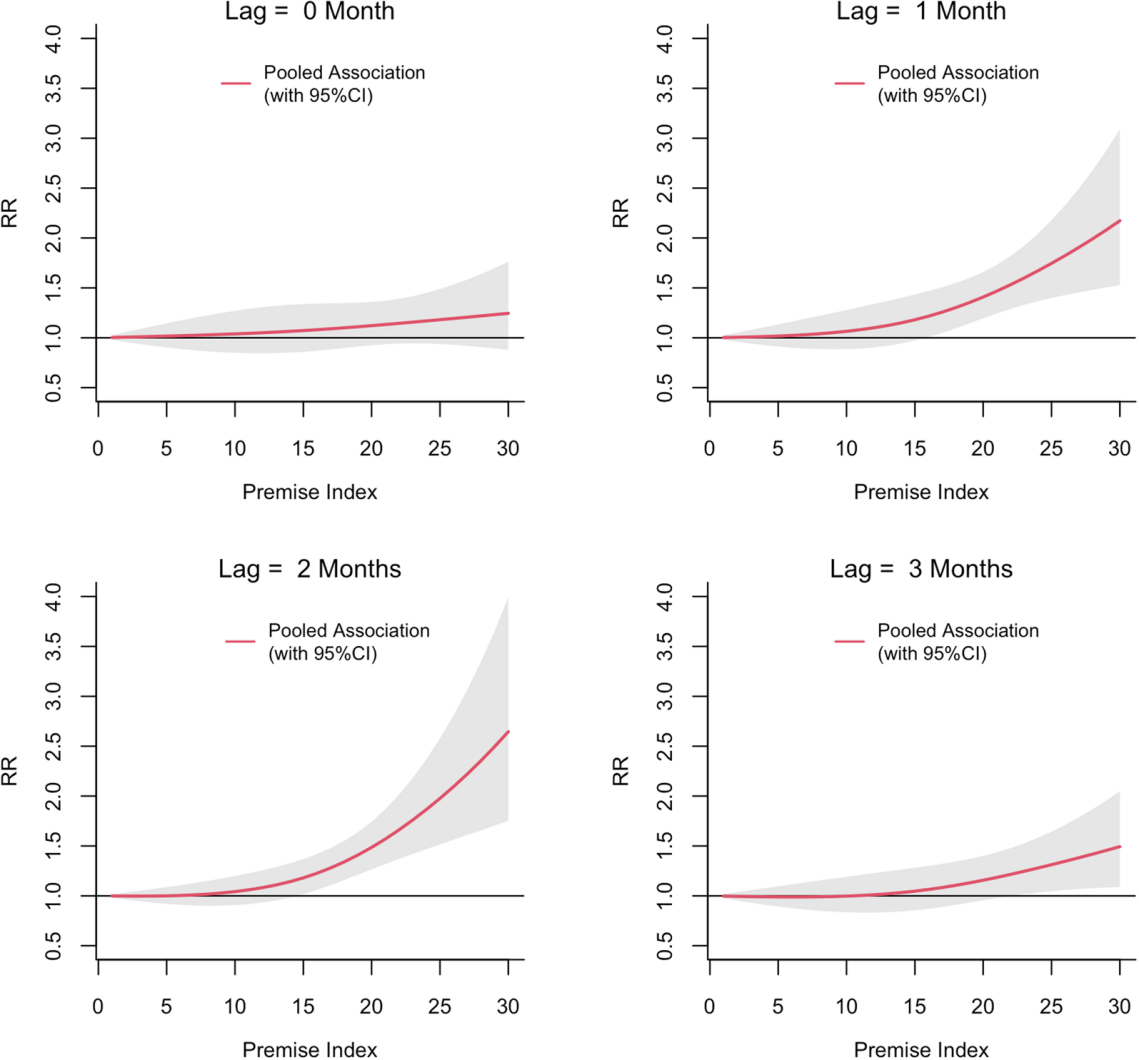
The pooled relative risk (RR) of dengue by PI at lag 0–3 months in ten MOH divisions in Kalutara district, Sri Lanka, 2010–2019. The *Y*-axis represents the RR for dengue incidence, with a RR of 1.0 at a PI of 0. The shaded areas in gray represent the 95% confidence intervals (CI)

**Fig. 4:**
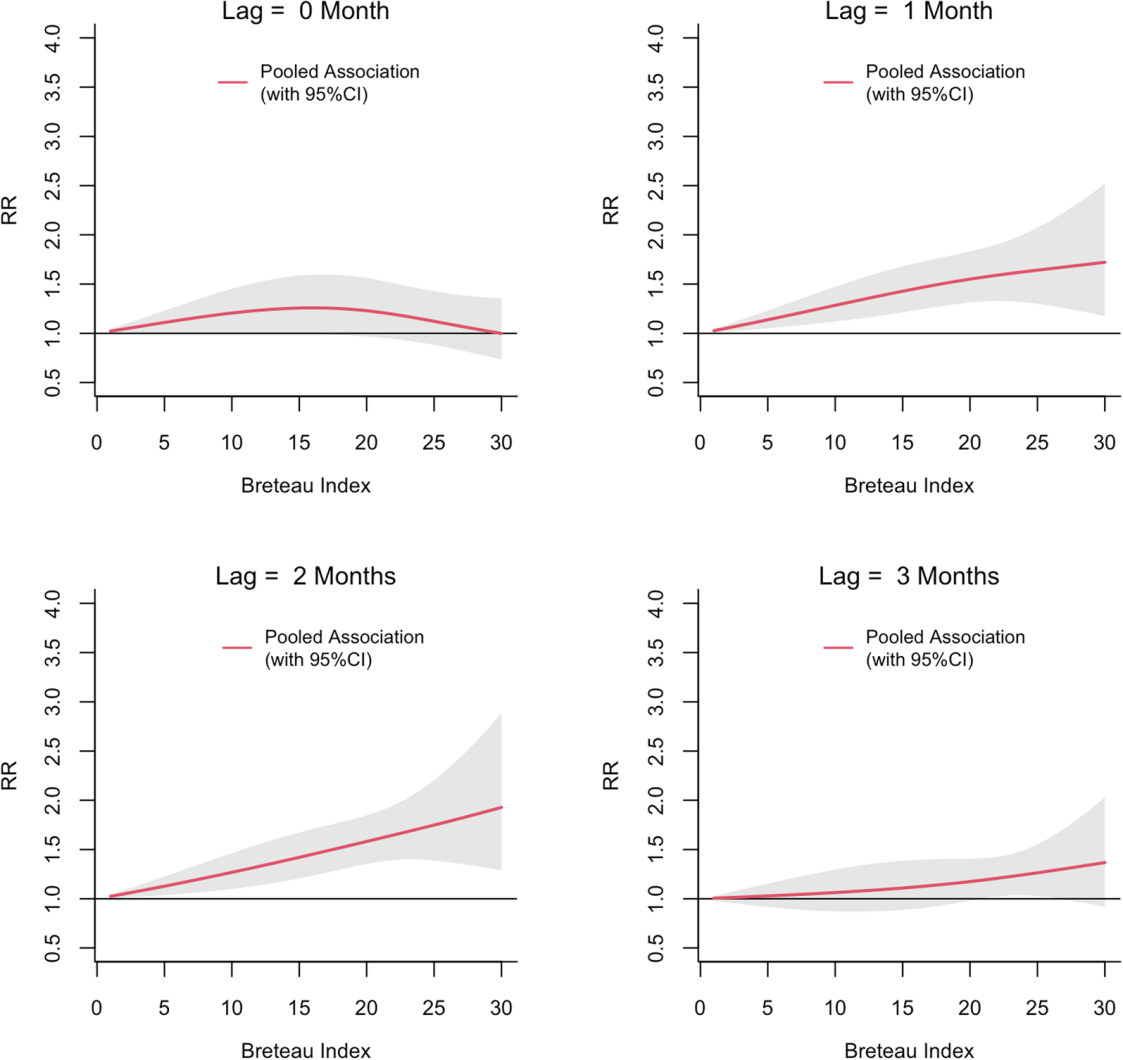
The pooled RR of dengue by BI at lag 0–3 months in Kalutara district, Sri Lanka, 2010–2019. The* Y*-axis represents the RR for dengue incidence, with a RR of 1.0 at a BI of 0. The shaded area in gray represents the 95% CI

**Fig. 5:**
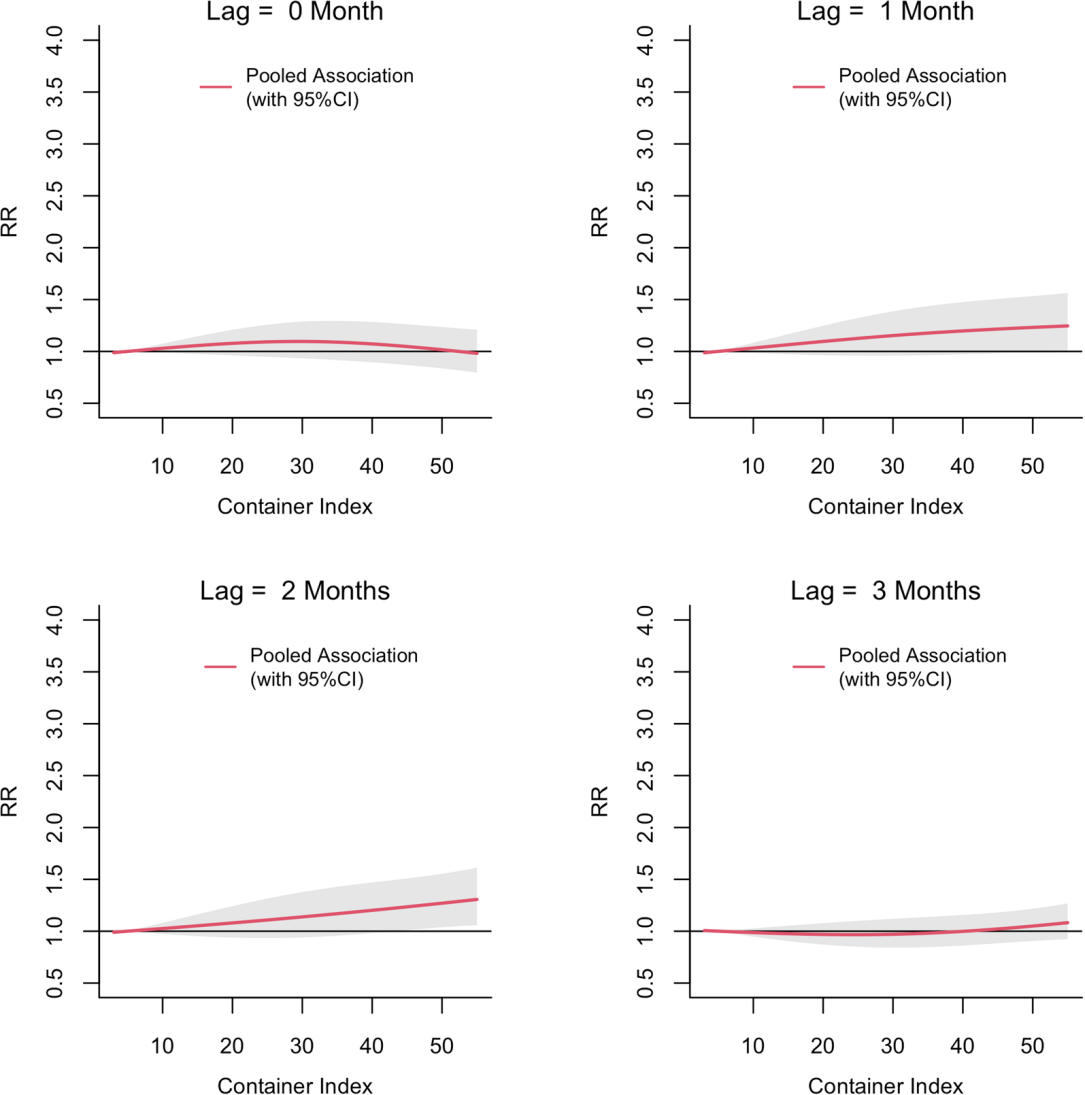
The pooled RR of dengue by CI at lag 0–3 months in Kalutara district, Sri Lanka, 2010–2019. The *Y*-axis represents the combined RR for dengue incidence, with a RR of 1.0 at a CI of 5. The shaded areas in gray represent the 95% CI

The exposure-lag-response associations between PI, BI and CI and dengue risk in each MOH division pooled and predicted at different lags are shown in Figs. 3, 4 and 5, respectively. The relative risk (RR) for dengue started to increase with a lag of 1 month for all three vector indices and then appeared to be decreasing after 3 months. The RR increased with increasing values of each index reaching a maximum at a lag of 2 months. The threshold value for PI for a statistically significant increase in the RR was 15. The BI seemed to have a lower threshold value of 2. The highest RR (2.64; 95% confidence interval [(CI] 1.75–3.99) was observed at a PI of 30 at a lag of 2 months. For a BI of 30, the RR was 1.92 (95% CI 1.28–2.88). The RR for CI also appeared to increase linearly with increasing lag period, but the association was statistically significant only at a lag of 2 months when CI > 45 (Fig. 5). The maximum RR observed for CI was 1.30 (95% CI 1.05–1.61).

The Cochran Q-test of heterogeneity was not significant for the PI (25.16, *df* = 18, *P*-value = 0.121), indicating a homogeneous PI to dengue relationship across all 10 MOH divisions (Additional file 1: Table S2). In contrast, the test statistics revealed that the heterogeneity of the vector indices and dengue association across MOH divisions were statistically significant for BI (31.01, *df* = 18, *P*-value = 0.029) and CI (34.09, *df* = 18, *P*-value = 0.012). As indicated by the *I*^2^ statistics, 41.95% of the variability of BI to dengue and 47.19% of the variability of CI to dengue associations could be attributed to true differences in the MOH division-specific variables. Among the variables analyzed, Wald test statistics derived through univariable multivariate meta-regression suggests that huts and shanties, number of schools and size of school-going population contributed significantly to the heterogeneity among divisions, thus modifying the BI-dengue relationship (Additional file 1: Table S3). For CI, temperature, household land use and number of households were the significant contributors (Additional file 1: Table S4). None of the variables contributed to the PI-dengue relationship, as indicated by the non-significant results for the Q-test and Wald test (Additional file 1: Table S2; Figure S4). We observed that huts and shanties at the 75th percentile of the range (*n* = 83) increased the pooled (district average) RR of dengue estimated by BI (Additional file 1: Table S3; Figure S5). Huts and shanties also seemed to explain a substantial amount of heterogeneity in the BI and dengue association among MOH divisions (*I*^2^ = 31.37%) compared to the base model with no predictors (*I*^2^ = 41.95%). Furthermore, the *Q*-test for the residual amount of heterogeneity was no longer significant (*P*-value = 0.106). Similarly, higher number of schools (*n* = 55) and larger school-going population (*n* = 26,376) appeared to increase the RR of dengue. High temperature (31.8 °C) seemed to increase the RR predicted by CI throughout the range of CI values. Here the effect of temperature was substantial, explaining a considerable amount of heterogeneity (*I*^2^ = 29.86%) compared to the base model (*I*^2^ = 47.19%). Household land use at its 75th percentile (1.1 ha per house) seemed to increase the RR of dengue while number of households (*n* = 37,956) seemed to decrease it (Additional file 1: Table S4; Figure S6).

Even though the moderating effect was not statistically significant (except for the variables described above), the following division-specific variables appeared to change the overall cumulative RR predicted by the base model. Higher rainfall (356.4 mm/month) and higher temperature (31.8 °C) appeared to increase the risk of dengue at increasing values of each of the three vector indices (Additional file 1: Figures S4–S6). Variation in population density did not seem to change the risk predictions by any of the vector indices. Furthermore, we observed a paradoxical decrease in the risk prediction using CI for the 75th percentile values of number of schools, school-going population, luxury houses, huts and shanties and manufacturing industries compared to increased risk predicted using BI. Large land areas and large household and per-capita land use appeared to increase the risk predicted using CI. Increasing area of rubber cultivation lands shifted the exposure–response curve above the district average once predicted by BI.

Overall, for all MOH divisions, a lag value of 1–2 months for each of the three vector indices showed low to moderate accuracy (area under the curve [AUC]: 0–70%) in predicting dengue epidemics (Table 2). A pooled analysis of which of the three indices best predicted dengue outbreaks showed that BI had superior overall predictive performance (62.8%; 95% CI 53.6–72.0%). For PI and BI, the predictive capacity appeared to be higher in rural MOH areas (Agalawatta, Bulathsinhala, Ingiriya, Palindanuwara and Madurawala) compared to urban areas (Panadura, Bandaragama and Horana). Although CI appeared to have the lowest overall predictive accuracy, in Horana, Bandaragama and Walallavita MOHs, this index demonstrated a higher predictive capacity compared to BI and PI.

**Table 2:** Performances of the area under the receiver operating characteristic curves of each vector index for outbreak years analyzed by Medical Officer of Health division and overall, for all the divisions in the Kalutara district

MOH Division	Premise Index (95% CI)	Breteau Index (95% CI)	Container Index (95% CI)
Agalawatta	55.7% (25.9–85.6)	66.7% (37.5–95.8)	38.0% (6.69–69.3)
Bandaragama	54.0% (24.9–83.6)	50.7% (25.9–85.6)	58.5% (26.3–75.0)
Bulathsinhala	52.5% (20.1–84.9)	69.4% (43.7–94.9)	61.0% (22.3–99.7)
Horana	49.5% (22.5–76.4)	62.7% (32.8–92.6)	66.1% (39.9–92.2)
Ingiriya	71.0% (39.9–100)	73.1% (42.3–100)	60.7% (23.1–98.2)
Madurewala	56.1% (25.6–86.6)	58.0% (30.3–85.7)	46.4% (17.9–74.8)
Matugama	50.0% (24.9–75.1)	66.6% (36.5–96.6)	43.2% (16.5–69.9)
Palindanuwara	55.8% (23.6–87.9)	71.2% (38.5–100)	49.1% (19.6–78.6)
Panadura	54.5% (20.1–88.8)	60.2% (30.9–89.4)	57.1% (19.2–95.0)
Wallavita	53.4% (13.7–93.1)	51.7% (12.5–90.9)	71.5% (45.5–97.5)
Overall for all divisions^a^	54.8% (45.2–64.5)	62.8% (53.6–72.0)	55.4% (46.0–64.7)

Data are presented as the percentage of area under the receiver operating characteristic curve for the Premise, Breteau, and Container indices for each MOH division pooled across all outbreak years, with the95% confidence interval (CI) in parentheses

^a^Overall performances for all MOH divisions

## Discussion

The lagged relations between the three vector indices and dengue were constant, and the strongest association was observed at a lag of 2 months. When vector indices exceeded their respective thresholds (as calculated here: BI = 2, PI = 15 and CI = 45), the relative risk of dengue increased following a lag period of 1 month, reaching its maximum in 2 months, and then subsiding. This 1- to 2-month lag would cover approximately 7–9 days of the larval development period [44], 3 days of searching for the first blood meal after emergence as an adult [45], 7–12 days of the incubation period of the virus inside the mosquito (the extrinsic incubation period) [46], 3–10 days of the incubation period within the human body before manifestation of the disease (intrinsic incubation period) [47] and the time taken to seek health care and be notified as a dengue case. It has also been reported that a 1-month lag period observed between increasing larval index values and dengue cases may also be possible in high transmission settings with suitable environmental conditions and short generation times [48]. Similar lagged associations between dengue and vector indices have been identified in different locations in Sri Lanka and other dengue-affected countries [16, 23, 25].

It is important to identify a pragmatic vector index threshold and the best index to capture that threshold in the process of setting up vector control implementation targets [14]. When pooling the associations of all the MOH divisions in Kalutara district, we found that BI had the lowest threshold among the three indices. A statistically significant risk of dengue was observed when BI ≥ 2, and the risk increased linearly with increasing index values. A BI threshold of 5 has been the general guideline for indicating an increased dengue outbreak risk at many study locations [12, 49] despite these values having been calculated for yellow fever decades ago [8, 10, 12]. A statistically significant threshold for CI was observed only at a lag of 2 months (CI = 45), which was at the higher end of the range identified in our study. Furthermore, a recent survey conducted in Colombo and Kandy districts in Sri Lanka found similar BI threshold values (BI = 2.4 for Colombo and BI = 3 for Kandy) and lower values for CI (CI = 5.5 for Colombo and CI = 6.9 for Kandy) when calculated for *Ae. aegypti* [22, 23]. In Thailand, vector density thresholds for dengue outbreak risk have been set at PI > 10, BI > 50 and CI > 1 [17]. In Singapore, an even lower threshold for dengue outbreaks was observed when the national overall PI was < 1% [27]. A lower critical threshold observed for BI compared to PI for the same range of values (0 to 30) may indicate a higher sensitivity of BI as an index. The relationship between CI and dengue was observed to be weak in the present study and in other studies conducted in Sri Lanka and other settings [16, 22, 23]. The threshold values of each vector index we found in Kalutara district support the growing body of evidence for their spatial variability associated with dengue risk in different regions of the world [25, 50–52].

PI had the most homogeneous vector-dengue association of the three larval indices and was not sensitive to any of the division-specific variables analyzed. BI was sensitive to number of schools, size of school-going population and presence of huts and shanties. The divisions with a larger school-going population and slums or huts (Panadura, Mathugama and Horana MOH divisions) appeared to be at a higher risk of dengue once BI exceeded 20. These divisions represented relatively urban settings in the district. In comparison, the CI-dengue association showed a higher heterogeneity and was sensitive to temperature and households with large land areas. High temperature and prevalence of premises with extensive land appeared to increase the risk of dengue at any CI value. Rural MOH divisions, such as Bulthsinhala, Palindanuwara and Walalavita, with low population densities and high per capita land use were likely to experience a higher risk with increasing temperature. We observed that municipality services were not widely available in these divisions. Therefore, a lack of waste management programs might have led to a higher prevalence of infested containers. This observation could lead to the paradoxical positive influence of increasing CI on the RR of dengue in areas with lower population densities and higher per capita land use. However, these findings may not be directly related to the division-specific factors analyzed. The moderating variables described above may represent various combinations of factors that may influence dengue risk, including human mobility, which we did not include in our models. We found that the mobility restrictions imposed to prevent the spread of COVID-19 reduced the risk of dengue among the school-going population in Sri Lanka, indicating the important role of human mobility when defining the dengue risk [53].

We observed that BI, which had the lowest threshold value, also demonstrated a superior overall predictive capacity compared to the other two indices. PI and CI were similar but had lower predictive capacity. Similar findings were observed for BI calculated for *Ae. aegypti* in a study conducted in two different dengue endemic districts in Sri Lanka [23]. A potential reason for BI being superior to other two indices in predicting dengue may be that, by definition, the index relates vector breeding sites more closely with human dwellings [10, 12]. However, when considering individual divisions, a wide variation in predictive capacity was observed. The highest predictive capacity for all three vector indices was observed for rural and low endemic divisions. The predictive capacity was lower for all indices in the highly endemic and highly urban Panadura, Horana, and Bandaragama MOH divisions. In the rural and less endemic divisions, the vector indices and their associations with dengue may be more stable due to fewer source reduction interventions and more localized outbreaks. In highly endemic settings, however, more complex transmission patterns may be observed due to more frequent and intense implementation of integrated vector management interventions, altered human-vector contact patterns and the complex nature of human mobility, all of which may obscure the association between vector indices and dengue incidence. Therefore, the predictive capacity of vector indices is time-bound, varies place to place and may be influenced by the effectiveness of vector control interventions, human mobility and population immunity due to previous infections. The predictive capacity of the larval indices was questioned in the settings where effective vector control interventions were in place and which subsequently had very low values of vector indices [16, 27].

An extensive source reduction program conducted with community and military participation (Civil-Military Corporation [CIMIC]) implemented in Panadura MOH division from 2014 to 2016 is an example of effective and cost-effective public health intervention. This intervention suppressed BI towards the threshold of 2, thereby averting about an estimated 50% of the dengue cases [54]. The study showed that vector control interventions, when implemented rigorously and well-coordinated, can be both effective and cost-effective in suppressing both BI and dengue incidence [54]. The authors also suggested that vector control interventions should be initiated with a lead time of at least 2 months [54], supporting our present observation of a 2-month lag between vector indices and dengue incidence. As such, a BI < 2 would be a tangible target, and importantly, it should always be sustained across all MOH divisions in Kalutara district.

We used combined vector indices in our study due to the low prevalence of *Ae. aegypti* in all MOH divisions. Because of this, a comparison of vector indices calculated for each of the two species and an analysis of their association with dengue incidence could not be achieved. However, using a combined index as a proxy for vector breeding where the dominant species is *Ae. albopictus* potentially captures the overall prevalence of *Aedes* larval stages as both species share similar breeding places. Furthermore, combined vector indices are more representative than each index used separately for all MOH divisions in the district, providing a rational basis for vector control decision-making by the epidemiologists and administrators of Kalutara district. The estimated thresholds in this empirical study depended on the values of vector indices and dengue incidence observed over the defined study period in each MOH division and are subject to change. The evaluation of their utility in predicting dengue risk was limited to the outbreak years. The 1- or 2-month lag identified in the present study appears to be similar to lags estimated by local weather variables and dengue [18]. Therefore, it may be valid to question whether vector index-based lead times would provide additional value. We believe that vector surveillance and *Aedes* larval indices are integral components of area-specific risk assessments, which facilitate the identification of vulnerable areas for prioritization of resources. In addition to providing quantifiable measures to assess implementation of interventions, vector surveillance has the additional value of providing information on the distribution of different breeding places and the productivity of these breeding places in time and space. Therefore, climate-based early warnings, coupled with environmental and vector surveillance information, have the potential to assist policy-makers in setting long-term, intermediate and short-term interventions along with more specific behavior change interventions in the community [55]. The value of *Aedes* larval indices as a proxy measure of adult vector densities has been questioned in many settings, and the utility of adult vector surveillance over simple larval surveys has been emphasized [13, 56]. The lack of scientific evidence showing statistically significant associations between larval indices and dengue is mainly because of the nature of the data and the methods used to date [10, 16]. The focus of our study was to generate scientific evidence on the association between larval indices and dengue incidence using data collected longitudinally and systematically in many locations and employing advanced and appropriate statistical methods. In addition, when considering programmatic perspectives, larval surveys enable rapid risk assessment as they are easy to perform and less time-consuming and resource-demanding [15]. Although adult vector surveillance is time-consuming and labor-intensive when conducted for selected areas, it could provide additional information on adult vector densities (adult PI, adult density and resting ratio) and behaviors (resting, dispersal and feeding). Such information would further inform targeting of rapid outbreak control interventions, such as fumigation and indoor residual spraying, that aim to reduce adult mosquito populations and thereby reduce the probability of human-vector contact [57]. Extraction of RNA and detection of DENV in adult mosquitoes have been shown to improve risk prediction and have been found to be a valuable addition to the existing *Aedes* vector surveillance tools [17, 58].

Based on the findings of this study, we propose a BI < 2 as the target for public health source reduction interventions in Kalutara district as this index was the most sensitive and predictive of an outbreak so far. BI in combination with meteorological data will further improve predictive performances and is increasingly being used in dengue forecast models with successful results [59]. Threshold-based location-specific forecasts with a lead time of 2 months, along with information on productive breeding places, would facilitate the implementation of rapid source reduction interventions. Heavy reliance on fixed vector index thresholds for long-term use is not recommended, and the thresholds need to be frequently evaluated using recent entomological and epidemiological data. The statistical model we developed can and should be updated with new data to generate the most reliable thresholds for the context in which they are being used. How frequently such updates should be done will depend on epidemiological and environmental factors and, most importantly, on the intensity of vector control interventions. As we observed, these thresholds vary considerably across study locations [16]. The results of our study may not be generalizable to all settings because the ecological and epidemiological variation is a fundamental feature of *Aedes* population and DENV transmission dynamics [60]. Therefore, it is important to understand location-specific relationships between *Aedes* vectors and dengue to be able to plan and implement effective vector control interventions. If long-term vector and disease surveillance information is available, the statistical framework we propose can be replicated in any setting to obtain robust and location-specific threshold estimates for vector indices to predict dengue transmission risk. The recently adopted Global Vector Control Response by the WHO highlighted the importance of enhancing vector surveillance for effective, locally adapted and sustainable interventions across sectors and vector borne diseases [61].

Even though the exposure–response association we found was not prominent, the value of CI in terms of monitoring and evaluation of source reduction programs cannot be underestimated. Information on types of containers or breeding sites should trigger a cascade of inter-sectoral and behavioral interventions to achieve a sustainable removal process. The positive moderating effect of high temperatures on CI and its association with dengue further highlight the increasing challenges for vector control in a warming world. Early warning systems combined with recommendations for effective vector surveillance and control interventions will be increasingly important to combat the additional disease burden associated with climate change [62]. Furthermore, when considering the severe dengue outbreaks in 2017 and 2019, we note that the vector indices can predict the risk but not the magnitude of the epidemic.

The moderating effect of the division-specific variables has the potential to inform a composite risk index for each division. Divisions with a high number of schools, huts and shanties and a large school-going population should be given priority. Selected premises such as schools should be attended frequently as they are important for having abundant mosquito breeding sites and for protecting students who are vulnerable with lower acquired immunity [53, 63, 64]. However, the utility of such division-specific variables should be further evaluated using appropriate study designs to provide more robust evidence. Vector control interventions with clearly defined monitoring and evaluating targets synergistically specified by *Aedes* vector indices are needed to reduce the dengue incidence by 25% as proposed by the WHO in the Global Strategy for Dengue Prevention and Control, 2021–2030 [65].

## Conclusions

*Aedes* larval indices, along with past and present disease trends, human mobility, climate factors and other division-specific factors, can readily be assimilated into the existing framework of public health vector control policy in Sri Lanka. Vector surveillance should be further strengthened, and systematic collection and reporting of surveillance data should be encouraged. Implementation targets should be set dynamically to guide effective and cost-effective source reduction programs to maintain vector indices below their threshold values. The methods we described here can be used to identify comparable lagged threshold values between *Aedes* larval indices and dengue incidence in any district of Sri Lanka and beyond, enabling policy-makers in affected countries to design evidence-based, holistic, and sustainable dengue vector control interventions.

## Supplementary Information

**Additional file 1: Text S1 **, **Table S1**. Definition of cross-basis function for the first- stage division specific models. **Figures S1–S3**. Model diagnostic plots for the PI, BI and CI, respectively. **Text S2**. Evaluation of the effect modification by the MOH division level factors derived from the second stage univariate meta-analysis. **Tables S2–S4.** Cochran Q-test of heterogeneity and related* P*-value along with *I*^2^ statistics and AIC and BIC obtained for the PI, BI and CI, respectively. **Figures S4–S6**. Moderating effect of division-specific variables on the overall cumulative exposure–response association between the PI, BI and CI, and dengue incidence, respectively. **Text S3.** Detailed methodology on evaluating the capacity of *Aedes* larval indices in predicting dengue outbreaks in Kalutara district.

## Data Availability

Dengue disease surveillance data are publicly available and can be accessed through the official website of the Epidemiology Unit, Ministry of Health Sri Lanka. (http://www.epid.gov.lk/web/index.php?option=com_casesanddeaths&Itemid=448&lang=en). *Aedes* vector surveillance and additional disease surveillance data that support the findings of this study are available from the Regional Director of Health Services Kalutra, but restrictions apply to the availability of these data that were used under license for the current study, and so are not publicly available. Data are however available from the authors upon reasonable request and with permission of National Dengue Control Unit, Ministry of Health Sri Lanka.

## Abbreviations

AIC: Akaike Information Criteria
BI: Breteau Index
CI: Container Index
DENV: Dengue virus
DLNM: Distributed lag non-linear models
HEO: Health Entomology Officers
MOH: Medical Officer of Health
PI: Premise Index
SIM: Second inter-monsoon
SWM: Southwest monsoon
