# Supplementary material for: Assessing the associations between Aedes larval indices and dengue risk in Kalutara district, Sri Lanka: a hierarchical time series analysis from 2010 to 2019
Source: Parasit Vectors. 2022 Aug 3;15:277. doi: 10.1186/s13071-022-05377-6 (PMC9351248; doi:10.1186/s13071-022-05377-6)
Supplement: Supplementary file 1 — Additional file 1: Text S1 , Table S1. Definition of cross-basis function for the first- stage division specific models. Figures S1–S3. Model diagnostic plots for the PI, BI and CI, respectively. Text S2. Evaluation of the effect modification by the MOH division level factors derived from the second stage univariate meta-analysis. Tables S2–S4. Cochran Q-test of heterogeneity and related P-value along with I2 statistics and AIC and BIC obtained for the PI, BI and CI, respectively. Figures S4–S6. Moderating effect of division-specific variables on the overall cumulative exposure–response association between the PI, BI and CI, and dengue incidence, respectively. Text S3. Detailed methodology on evaluating the capacity of Aedes larval indices in predicting dengue outbreaks in Kalutara district. [file 13071_2022_5377_MOESM1_ESM.docx]

**Additional Information**

Table of Contents

[Text S1: Definitions of the cross-basis function for the first-stage division specific analysis 2](#_Toc97459820)

[Table S1: Definitions of cross basis functions and sum of quasi-AIC calculated for the first stage dengue-vector models 2](#_Toc97459821)

[Figure S1: Model diagnostic plots showing the residual versus fitted values, normal Q-Q plot, scale location, residual versus leverage, histogram of residuals and partial auto correlation function for the Premise index. 3](#_Toc97459822)

[Figure S2: Model diagnostic plots showing the residual versus fitted values, normal Q-Q plot, scale location, residual versus leverage, histogram of residuals and partial auto correlation function for the Breteau index. 3](#_Toc97459823)

[Figure S3: Model diagnostic plots showing the residual versus fitted values, normal Q-Q plot, scale location, residual versus leverage, histogram of residuals and partial auto correlation function for the Container index. 4](#_Toc97459824)

[Text S2: Evaluation of the effect modification by the MOH division level factors derived from the second stage univariate meta-analysis 5](#_Toc97459825)

[Table S2: Cochran Q-test of heterogeneity and related p value along with I2 statistics and AIC obtained for Premise Index in second staged multivariate meta-analysis from 2010 to 2019 in 10 MOH division in Kalutara. 5](#_Toc97459826)

[Table S3: Cochran Q-test of heterogeneity and related p value along with I2 statistics and AIC and BIC obtained for Breteau Index in second staged multivariate meta-analysis from 2010 to 2019 in 10 MOH division in Kalutara. 6](#_Toc97459827)

[Table S4: Cochran Q-test of heterogeneity and related p value along with I2 statistics and AIC and BIC obtained for Container Index in second staged multivariate meta-analysis from 2010 to 2019 in 10 MOH division in Kalutara. 7](#_Toc97459828)

[Figure S4: Moderating effect of division-specific variables on the overall cumulative exposure-response association between Premise Index and dengue incidence from 2010 to 2019 in Kalutara district. 8](#_Toc97459829)

[Figure S5: Moderating effect of division-specific variables on the overall cumulative exposure-response association between Breteau Index and dengue incidence from 2010 to 2019 in Kalutara district. 9](#_Toc97459830)

[Figure S6: Moderating effect of division-specific variables on the overall cumulative exposure-response association between Container Index and dengue incidence from 2010 to 2019 in Kalutara district. 10](#_Toc97459831)

[Text S3: Evaluating the capacity of Aedes larval indices in predicting dengue outbreaks in Kalutara district 11](#_Toc97459832)

[Reference 12](#_Toc97459833)

# **Text S1: Definitions of the cross-basis function for the first-stage division specific analysis**

A flexible cross-basis function was defined using a natural cubic spline for each larval index allowing one degree of freedom with common internal and boundary knot placement for all the MOH divisions. The internal knots were placed at 15, 20, and 30 for PI, BI, and CI, respectively. The boundary knots for each Aedes lraval index were placed at values corresponding to the average minimum and maximum for all MOH divisions. This common knot position ensures the estimate coefficient to have the same interpretation across divisions. The central reference value of the cross-basis function was set at 0 for PI and BI and 5 for CI. The relative risk was estimated with reference to the risk at these centering values for the corresponding larval index. We incorporated a lag period of 0 to 3 months to cover the possible time segments of the mosquito life cycle, the generation time (time elapsed from one cycle of vector-to-human transmission and to the start of a new cycle) [1] and the notification time (time from the date of diagnosis to the date of notification to the public health system). The lag space in the cross-basis function was defined with two degrees of freedom with an internal knot at a lag of two months. Natural cubic spline function to the time variable was used in the model to account for time varying confounders and long-term trend. Here we used two degree of freedom per year considering the two season peaks in dengue in each year. We conducted a sensitivity analysis by changing different internal knot combinations when defining the cross-basis function in first stage models. The fit of the models were evaluated by calculating the quasi-Akaike Information Criteria (AIC) [2]. The best-fit model in each larval index had the minimum value of the sum of AIC in all ten MOH divisions. We further examined the residual diagnostics plots and partial-autocorrelation function during the evaluation.

## **Table S1:** Definitions of cross basis functions and sum of quasi-AIC calculated for the selected first stage dengue-vector models

| **Larval Index** | **Spline Function** | **Degree of freedom** | **Internal knot placement** | **Reference value** | **Sum of quasi-AIC** |
| --- | --- | --- | --- | --- | --- |
| Premise Index | Natural cubic spline | 1 | 15 | 0 | 12379.19 |
| Breteau Index | Natural cubic spline | 1 | 20 | 0 | 12175.04 |
| Container Index | Natural cubic spline | 1 | 30 | 5 | 12592.49 |

Model diagnostic figures (Figures S1 to S3) for each index are shown below. All parameters shown suggest a reasonably good model fit for each larval index and dengue.

**Premise Index**


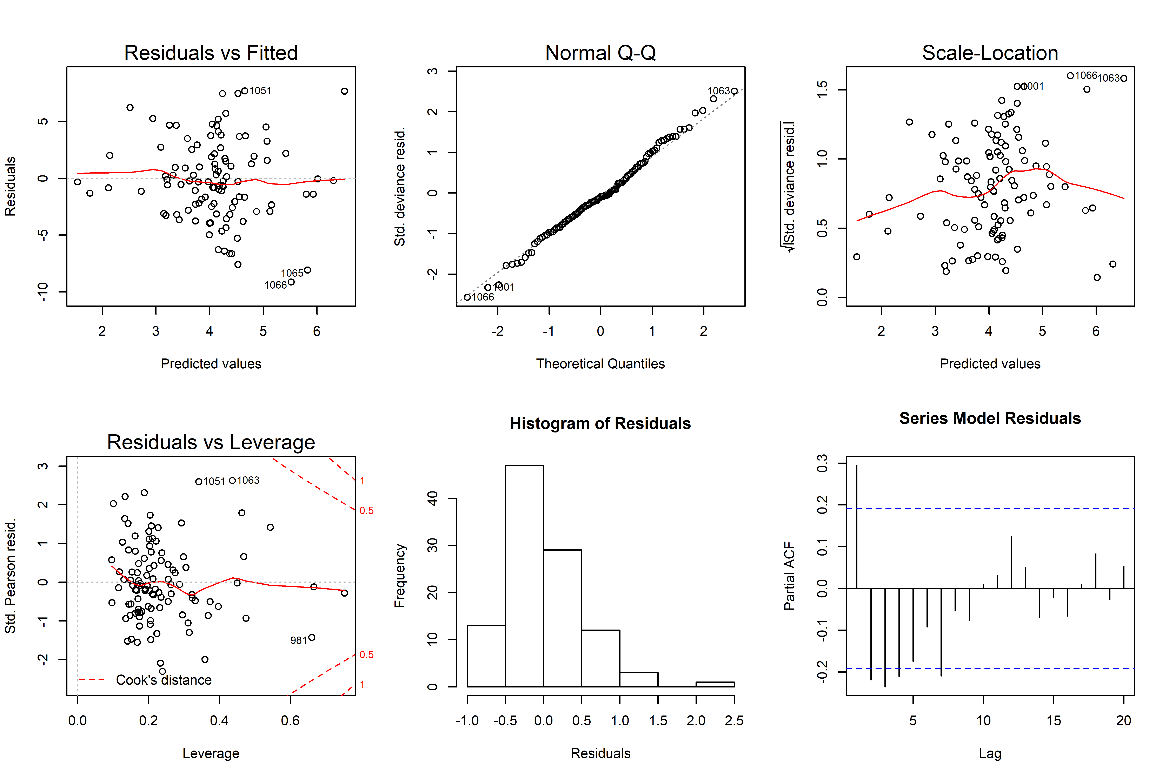


## **Figure S1:** Model diagnostic plots showing the residual versus fitted values, normal Q-Q plot, scale location, residual versus leverage, histogram of residuals and partial auto correlation function for the Premise index.

**Breateau Index**


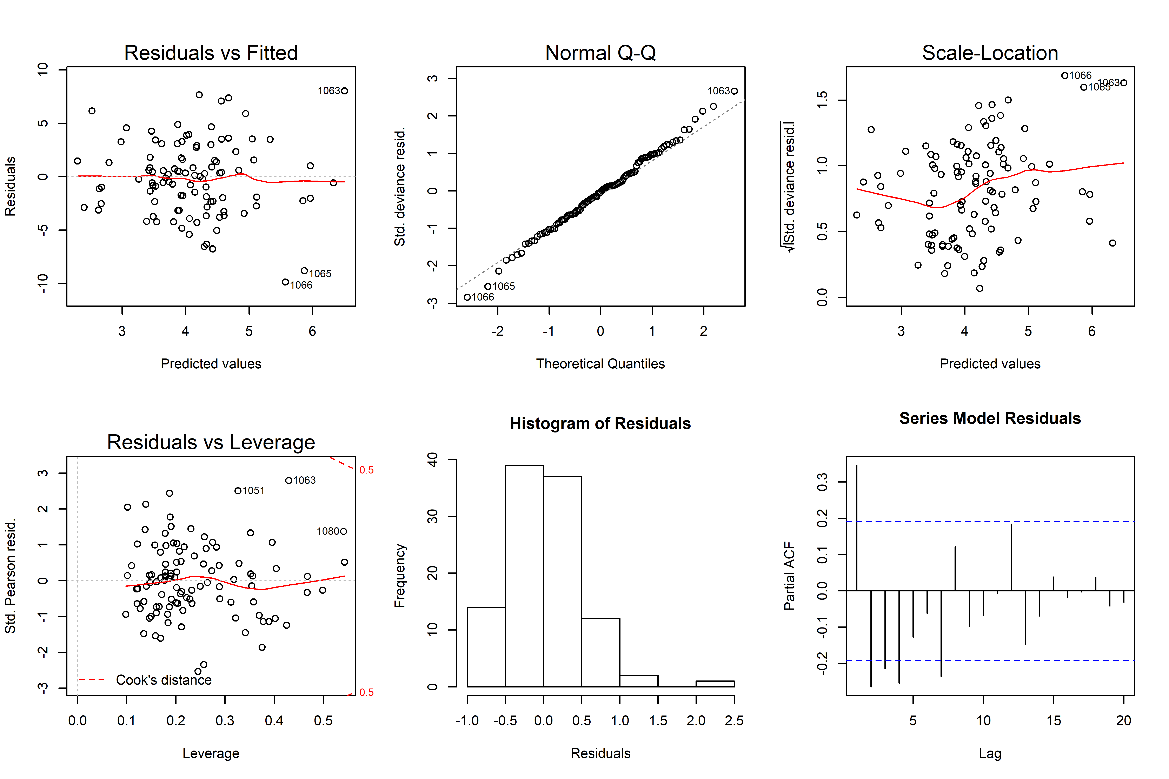


## **Figure S2:** Model diagnostic plots showing the residual versus fitted values, normal Q-Q plot, scale location, residual versus leverage, histogram of residuals and partial auto correlation function for the Breteau index.

**Container Index**


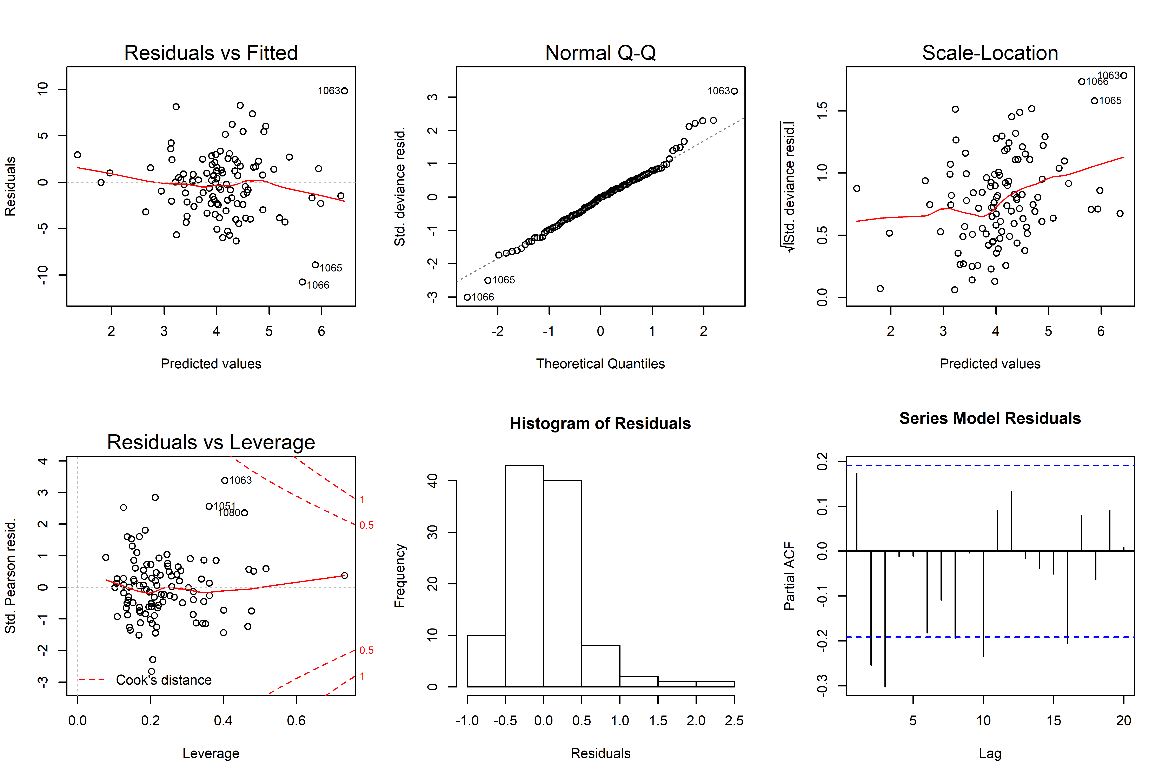


## **Figure S3:** Model diagnostic plots showing the residual versus fitted values, normal Q-Q plot, scale location, residual versus leverage, histogram of residuals and partial auto correlation function for the Container index.

# **Text S2: Evaluation of the effect modification by the MOH division level factors derived from the second stage univariate meta-analysis**

Univariable multivariate meta-regression framework was used to evaluate the moderator effect of each division specific variable at their 25^th^ and 75^th^ percentile values. The significant tests were done using an *p* value of 0.05 and a 95% confidence limit for all variables. The Table S2, Table S3 and Table S4 show the direction of moderator effect of each variable and the Wald test results for Premise, Breteau and Container Indices respectively. The Figure S4, Figure S5 and Figure S6 further demonstrate the direction of moderation predicted at 25^th^ and 75^th^ percentile values of each division specific variable with respect to ascending order of values of Premise, Breteau and Container Index respectively.

**Table S2:** Cochran Q-test of heterogeneity and related p value along with I2 statistics and AIC obtained for Premise Index in second staged multivariate meta-analysis from 2010 to 2019 in 10 MOH division in Kalutara. Results of the meta-regression of division specific variables explaining the heterogeneity with the corresponding Wald test of significance is shown.

| **Intercept only model and variables** | The direction of moderation at the 75^th^ percentile | **Cochran Q test** | | | **I-square** | Information criteria | **Wald test** | | |
| --- | --- | --- | --- | --- | --- | --- | --- | --- | --- |
|  |  | **Q** | **df** | **p** | **%** | **AIC** | **stat** | **df** | **p** |
| **Intercept only (Base model for PI)** |  | **25.16** | **18** | **0.121** | **28.45** | **83.33** | NA | NA | NA |
| Land area of rubber cultivation (hectare) | Ambiguous | 22.62 | 16 | 0.124 | 29.27 | 85.03 | 2.35 | 2 | 0.309 |
| Number of households | Increased risk | 23.45 | 16 | 0.102 | 31.76 | 85.13 | 2.2 | 2 | 0.332 |
| Temperature (C^0^) | Increased risk | 23.46 | 16 | 0.102 | 31.80 | 85.43 | 1.92 | 2 | 0.383 |
| Mean cumulative rainfall (mm/month) | Increased risk | 23.91 | 16 | 0.091 | 33.09 | 85.72 | 1.65 | 2 | 0.439 |
| Per-capita land use (hectare) | Ambiguous | 24.05 | 16 | 0.088 | 33.48 | 85.99 | 1.36 | 2 | 0.508 |
| Number of schools | Ambiguous | 23.81 | 16 | 0.094 | 32.81 | 86.05 | 1.28 | 2 | 0.526 |
| Household land use (hectare) | Ambiguous | 24.37 | 16 | 0.082 | 34.35 | 86.42 | 0.96 | 2 | 0.62 |
| Land area (hectare) | Ambiguous | 24.44 | 16 | 0.080 | 34.54 | 86.50 | 0.86 | 2 | 0.651 |
| Population density | Ambiguous | 24.72 | 16 | 0.075 | 35.26 | 86.48 | 0.85 | 2 | 0.652 |
| Huts and Shanties | Ambiguous | 24.81 | 16 | 0.073 | 35.50 | 86.66 | 0.67 | 2 | 0.714 |
| Luxury houses | Increased risk | 24.97 | 16 | 0.070 | 35.91 | 86.97 | 0.38 | 2 | 0.827 |
| School going population | Ambiguous | 25.04 | 16 | 0.069 | 36.09 | 87.20 | 0.13 | 2 | 0.936 |
| Manufacturing industry | Ambiguous | 24.80 | 16 | 0.073 | 35.49 | 87.22 | 0.11 | 2 | 0.947 |

**Table S3:** Cochran Q-test of heterogeneity and related p value along with I2 statistics and AIC and BIC obtained for Breteau Index in second staged multivariate meta-analysis from 2010 to 2019 in 10 MOH division in Kalutara. Results of the meta-regression of division specific variables explaining the heterogeneity with the corresponding Wald test of significance is shown.

| **Intercept only model and variables** | The direction of moderation at the 75^th^ percentile | **Cochran Q test** | | | **I-square** | Information criteria | **Wald test** | | |
| --- | --- | --- | --- | --- | --- | --- | --- | --- | --- |
|  |  | **Q** | **df** | **p** | **%** | **AIC** | **stat** | **df** | **p** |
| **Intercept only (Base model for BI)** |  | **31.01** | **18** | **0.029** | **41.95** | **83.77** | NA | NA | NA |
| Huts and Shanties | Increased risk | 23.31 | 16 | 0.106 | 31.37 | 81.79 | 7.28 | 2 | 0.026 |
| Number of schools | Increased risk | 24.14 | 16 | 0.086 | 33.73 | 83.51 | 6.47 | 2 | 0.039 |
| School going population | Increased risk | 25.38 | 16 | 0.063 | 36.97 | 83.29 | 6.03 | 2 | 0.049 |
| Mean monthly temperature (0C) | Increased risk | 26.68 | 16 | 0.045 | 40.04 | 85.25 | 4.03 | 2 | 0.133 |
| Land area (hectare) | Ambiguous | 29.27 | 16 | 0.022 | 45.35 | 86.15 | 1.76 | 2 | 0.412 |
| Household land use (hectare) | Ambiguous | 29.24 | 16 | 0.022 | 45.28 | 86.42 | 1.56 | 2 | 0.458 |
| Luxury houses | Increased risk | 29.2 | 16 | 0.023 | 45.21 | 86.42 | 1.43 | 2 | 0.489 |
| Number of households | Increased risk | 30.17 | 16 | 0.017 | 46.96 | 86.72 | 1.12 | 2 | 0.572 |
| Per-capita land use (hectare) | Ambiguous | 30.14 | 16 | 0.017 | 46.91 | 86.88 | 0.99 | 2 | 0.607 |
| Land area of rubber cultivation (hectare) | Increased risk | 28.79 | 16 | 0.025 | 44.42 | 87.18 | 0.86 | 2 | 0.652 |
| Mean cumulative rainfall (mm/month) | Increased risk | 29.61 | 16 | 0.02 | 45.96 | 87.02 | 0.83 | 2 | 0.66 |
| Manufacturing industry | Ambiguous | 28.92 | 16 | 0.024 | 44.68 | 87.29 | 0.71 | 2 | 0.701 |
| Population density | Ambiguous | 30.78 | 16 | 0.014 | 48.02 | 87.31 | 0.5 | 2 | 0.778 |

**Table S4:** Cochran Q-test of heterogeneity and related p value along with I2 statistics and AIC and BIC obtained for Container Index in second staged multivariate meta-analysis from 2010 to 2019 in 10 MOH division in Kalutara. Results of the meta-regression of division specific variables explaining the heterogeneity with the corresponding Wald test of significance is shown.

| **Intercept only model and variables** | The direction of moderation at the 75^th^ percentile | **Cochran Q test** | | | **I-square** | Information criteria | **Wald test** | | |
| --- | --- | --- | --- | --- | --- | --- | --- | --- | --- |
|  |  | **Q** | **df** | **p** | **%** | **AIC** | **stat** | **df** | **p** |
| **Intercept only (Base model for CI)** |  | **34.09** | **18** | **0.012** | **47.19** | **82.69** | NA | NA | NA |
| Temperature (C^0^) | Increased risk | 22.81 | 16 | 0.119 | 29.86 | 79.68 | 8.77 | 2 | 0.012 |
| Household land use | Increased risk | 26.45 | 16 | 0.048 | 39.50 | 81.31 | 6.17 | 2 | 0.046 |
| Number of households | Decreased risk | 29.29 | 16 | 0.022 | 45.37 | 80.73 | 6.12 | 2 | 0.047 |
| Population density | Ambiguous | 29.59 | 16 | 0.020 | 45.93 | 81.57 | 5.19 | 2 | 0.075 |
| Land area (hectare) | Increased risk | 27.42 | 16 | 0.037 | 41.66 | 82.39 | 4.76 | 2 | 0.093 |
| Per-capita land use (hectare) | Increased risk | 28.80 | 16 | 0.025 | 44.44 | 82.58 | 4.51 | 2 | 0.105 |
| School going population | Decreased risk | 29.84 | 16 | 0.019 | 46.38 | 83.00 | 4.34 | 2 | 0.114 |
| Mean cumulative rainfall (mm/month) | Increased risk | 29.68 | 16 | 0.020 | 46.09 | 82.76 | 4.03 | 2 | 0.133 |
| Luxury houses | Decreased risk | 30.58 | 16 | 0.015 | 47.68 | 83.03 | 3.66 | 2 | 0.160 |
| Huts and Shanties | Decreased risk | 31.74 | 16 | 0.011 | 49.60 | 84.50 | 2.36 | 2 | 0.307 |
| Number of schools | Decreased risk | 29.33 | 16 | 0.022 | 45.45 | 85.08 | 1.83 | 2 | 0.400 |
| Manufacturing industry | Decreased risk | 30.99 | 16 | 0.013 | 48.38 | 85.83 | 0.92 | 2 | 0.632 |
| Land area of rubber cultivation (hectare) | Ambiguous | 33.63 | 16 | 0.006 | 52.42 | 86.21 | 0.52 | 2 | 0.770 |

**Figure S4:** Moderating effect of division-specific variables on the overall cumulative exposure-response association between Premise Index and dengue incidence from 2010 to 2019 in Kalutara district. Each panel in the figure illustrates the moderating effect of the division-specific variable on the exposure-response association between Premise Index and dengue incidence. The Y axis indicates the relative risk (RR) estimate with reference to the Premise index of 0. The X axis indicates the Premise Index values. The moderating effect of values corresponding to the 25^th^ and 75^th^ percentiles of each division-specific variable is shown in blue and red dashed lines, respectively. The solid black line indicates the overall cumulative exposure-response association for the district (District Average).

**Figure S5:** Moderating effect of division-specific variables on the overall cumulative exposure-response association between Breteau Index and dengue incidence from 2010 to 2019 in Kalutara district. Each panel in the figure illustrates the moderating effect of the division specific variable on the exposure-response association between Breteau Index and dengue incidence. The Y axis indicates the relative risk (RR) estimate with reference to the Breteau Index of 0. The X axis indicates the Breteau Index values. The moderating effect of values corresponding to the 25^th^ and 75^th^ percentiles of each division-specific variable is shown in blue and red dashed lines, respectively. The solid black line indicates the overall cumulative exposure-response association for the district (District Average).

**Figure S6:** Moderating effect of division-specific variables on the overall cumulative exposure-response association between Container Index and dengue incidence from 2010 to 2019 in Kalutara district. Each panel in the figure illustrates the moderating effect of the division specific variable on the exposure-response association between Container Index and dengue incidence. The Y axis indicates the relative risk (RR) estimate with reference to the Container Index of 5. The X axis indicates the Container Index values. The moderating effect of values corresponding to the 25^th^ and 75^th^ percentiles of each division-specific variable is shown in blue and red dashed lines, respectively. The solid black line indicates the overall cumulative exposure-response association for the district (District Average).

# **Text S3: Evaluating the capacity of *Aedes* larval indices in predicting dengue outbreaks in Kalutara district**

Prediction models were developed in the generalized additive modelling (GAM) framework [3]. Quasi-Poisson distribution was selected to account for the overdispersion of data. Five models were developed for each larval index based on the lag values used. The first three were developed using single lagged values of 1, 2, and 3 months, and two models with combined lagged values consist of (lag 1 + lag 2) and (lag 1 + lag 2 + lag 3), respectively. Altogether fifteen models were tested upon the 10 MOH divisions for all three larval indices. Each model was evaluated in a 'leave one out' cross-validation mode for each year from January 2010 to December 2019 – that is, by excluding the year for which the prediction was made, and the model parameters were estimated. R-square and deviance explained were used to evaluate the yearly predictive performances in each model. Since the predictive performance was better for the model with the larval index with lagged values one and two months, that model was used for the prediction evaluation. Finally, three models were developed for three larval indices containing lagged values of one and two months each. The common formula is given below.

***Prediction model***

*D_i_ ~ quasiPoisson(µ_ti_)*

*E(D_(ti)_) = β_i_ + f(LI_it-1_, vardf) + f(LI_it-2_, vardf)*

Where *E(D_(ti)_)* is the expected number of dengue cases in month *t* in a MOH division denoted by *i*; *β* is the intercept in MOH division *i*; *f* is the natural cubic spline function for one and two months lagged values of each larval index (*LI)* in each *i* MOH division with corresponding degrees of freedom (*vardf*). Three models were developed using PI lag 1 + PI lag 2, BI lag 1 + BI lag 2, and CI lag 1 + CI lag 2, as predictor variables, denoted by *LI_it-1_* and *LI_it-2,_* respectively as in the common formula above. Monthly dengue cases were assumed to follow a quasi-Poisson distribution, which allows over-dispersion. The degree of freedom for the lagged larval indices was set at one per year for the total training period of nine years. The capacity of each larval index to predict outbreak months in MOH divisions with a lead time of one to two months was evaluated using the final prediction models.

So far, there is no consensus on defining dengue outbreak thresholds [4]. Therefore, a dengue outbreak year was defined operationally by the National Dengue Control Unit as one when the annual incidence of dengue exceeded 100 cases per 100,000 population in any given MOH division. We converted this yearly rate into a monthly rate for each MOH division to obtain monthly thresholds as cut-off values for outbreak prediction. For the observed series, in a given MOH division, the outbreak month was identified when the observed dengue cases in the particular month exceeding the corresponding monthly threshold value [5]. Months with predictions beyond these thresholds were taken as outbreak months for the prediction series. Binary variables with zero (no outbreak) and one (outbreak) were created for observed and predicted dengue cases for each month for each evaluation year. These binary variables were used to evaluate the yearly predictive performance with respect to sensitivity, specificity, positive predictive value, and area under the curve (AUC) in receiver operating characteristic curves (ROC) using R packages pROC [6]. The AUC values were then meta-analysed to pool across all the outbreak years to obtain a joint estimate for each larval index by MOH division. Generally, an AUC of 0.5 suggests no discrimination (i.e., ability to diagnose patients with and without the disease or condition based on the test), 0.7 to 0.8 is considered acceptable, 0.8 to 0.9 is considered excellent, and more than 0.9 is considered outstanding [7,8]. We applied this general rule in evaluating the predictive accuracy of Aedes larval indices in predicting dengue epidemics.

# **Reference**

1. Rocklöv J, Tozan Y. Climate change and the rising infectiousness of dengue. Emerg Top Life Sci. 2019;3: 133–142. doi:10.1042/etls20180123

2. Gasparrinia A, Armstrong B, Kenward MG. Distributed lag non-linear models. Stat Med. 2010;29: 2224–2234. doi:10.1002/sim.3940

3. Wood SN. Generalized additive models: An introduction with R, second edition. Generalized Additive Models: An Introduction with R, Second Edition. Chapman and Hall/CRC; 2017. doi:10.1201/9781315370279

4. Brady OJ, Smith DL, Scott TW, Hay SI. Dengue disease outbreak definitions are implicitly variable. Epidemics. 2015;11: 92–102. doi:10.1016/j.epidem.2015.03.002

5. Bowman LR, Tejeda GS, Coelho GE, Sulaiman LH, Gill BS, McCall PJ, et al. Alarm variables for dengue outbreaks: A multi-centre study in Asia and Latin America. Hsieh Y-H, editor. PLoS One. 2016;11: e0157971. doi:10.1371/journal.pone.0157971

6. Robin X, Turck N, Hainard A, Tiberti N, Lisacek F, Sanchez JC, et al. pROC: An open-source package for R and S+ to analyze and compare ROC curves. BMC Bioinformatics. 2011;12: 77. doi:10.1186/1471-2105-12-77

7. Mandrekar JN. Receiver operating characteristic curve in diagnostic test assessment. J Thorac Oncol. 2010;5: 1315–1316. doi:10.1097/JTO.0b013e3181ec173d

8. Greiner M, Pfeiffer D, Smith RD. Principles and practical application of the receiver-operating characteristic analysis for diagnostic tests. Prev Vet Med. 2000;45: 23–41. doi:10.1016/S0167-5877(00)00115-X
